# 104-week safety and effectiveness of dupilumab in the treatment of severe atopic dermatitis. The experience of 5 reference dermatology units in Spain^☆^^☆☆^

**DOI:** 10.1016/j.abd.2020.08.030

**Published:** 2021-09-27

**Authors:** Jose Juan Pereyra-Rodriguez, Javier Dominguez-Cruz, Jose Carlos Armario-Hita, Ricardo Ruiz-Villaverde

**Affiliations:** a Dermatology Unit, Hospital Universitario Virgen del Rocio, Sevilla, Spain; b Dermatology Unit, Hospital Universitario Puerto Real, Cádiz, Spain; c Dermatology Unit, Hospital Universitario San Cecilio, Granada, Spain

Dear Editor,

Atopic Dermatitis (AD) is a multifactorial disease resulting from the interaction of genetic predisposition, environmental triggers, disruption of skin barrier function, and type 2 immune dysregulation. Management of mild forms of AD includes the use of emollients, topical corticosteroids or calcineurin inhibitors, and phototherapy, while systemic immunosuppressive agents such as oral corticosteroids and Cyclosporine A (CsA) are reserved for severe refractory cases.^1^ Nevertheless, severe cases are usually not adequately controlled with any of these therapies, requiring a further step to reach clinical control.^2^ Recently, FDA and EMA have authorized Dupilumab, a treatment targeting Th2 cytokines Il-4 and Il-13 which has shown to be effective to control the signs and symptoms of AD. Real-world experience with Dupilumab shows a similar effectivity as compared to randomized clinical trials, but it is yet to know how this drug will perform in the long term in routine medical practice.^3^, ^4^, ^5^

We performed a retrospective chart review of 30 patients from 5 Andalusian reference dermatology units (Hospital Virgen del Rocio-Sevilla; Hospital Juan Ramon Jimenez-Huelva; Hospital Universitario de Puerto Real-Cadiz; Hospital Reina Sofía-Córdoba and Hospital Universitario San Cecilio-Granada) included in the Spanish compassionate use of Dupilumab for adult patients with moderate to severe AD from November 2017 to February 2020. According to the compassionate use program recommendations for dupilumab prescription, inclusion criteria were age ≥18 years, a severe disease defined by a baseline Eczema Area and Severity Index (EASI) ≥16, and inadequate response/intolerance to CsA or medical inadvisability of CsA treatment. Patients with any documented psychiatric comorbidity were excluded from the study.

All patients were treated with subcutaneous dupilumab 300 mg every other week following a loading dose of dupilumab 600 mg. Concomitant topical corticosteroids or calcineurin inhibitors were allowed. All patients agreed with the treatment regimen and signed a written consent form to extract relevant data from their charts. Data collected included age, disease course, personal history (including comorbidities), and previous systemic/biological treatments. Disease severity was measured by Scoring Atopic Dermatitis (SCORAD) and Pruritus Visual Analog Scale (VAS) scores at the baseline visit, and at follow-up weeks 4, 12, 24, 52, 76, and 104. Quality of life was assessed with Dermatology Life Quality Index (DLQI).

Baseline characteristics are shown in Table 1. The studied population presented a substantial burden of disease, with an average of 25.7 years of AD evolution. The most common comorbidities were allergic rhinitis (55.5%), asthma (37%), and conjunctivitis (33.4%). These comorbidities were more common in those patients with longer treatment with immunosuppressants. All patients had received previous treatment with oral corticosteroids, and 96.3% had been treated with cyclosporine.^3^ The effectiveness of the dupilumab treatment was assessed at weeks 4, 12, 24, 52, and 104. The mean percentage of change in SCORAD is shown in Fig. 1, while DLQI evolution is shown in Fig. 2. At baseline, SCORAD was 59.4, while pruritus VAS was 8.3. In the follow-up week 104 visit, SCORAD decreased to 10.7 (-82%), and pruritus VAS reduced to 2.9 (-65%). Regarding QoL, the baseline DLQI value was 19, reaching 4.3 (-77.4%) at the same cut-off. The safety profile was favorable, reporting 3 cases of mild conjunctivitis, managed positively without suspension of Dupilumab. One example of treatment effect is shown at baseline (Fig. 3) and 16 weeks (Fig. 4).

**Table 1:** Patients’ baseline characteristics.

Characteristic	Average
Age, years (range)	40.4 (19–56)
Sex (male, %)	70% (21/30)
Years of AD evolution, years (range)	28.5 (10–44)
Comorbidities (%)	
Nasal polyps	16.6%
Conjunctivitis	36.8%
Extrinsic asthma	22.2%
Allergic rhinitis	50%
Alimentary allergies	22.2%
Previous treatment	
Oral systemic corticosteroids	100% (Average length: 23.7 months)
Oral cyclosporine	94.4% (Average’s lengths: 19.8 months)
Phototherapy (NB-UVB)	33.3%
Baseline SCORAD	59.4%
Baseline pruritus VAS	8.3%
Baseline DLQI	19

**Figure 1:**
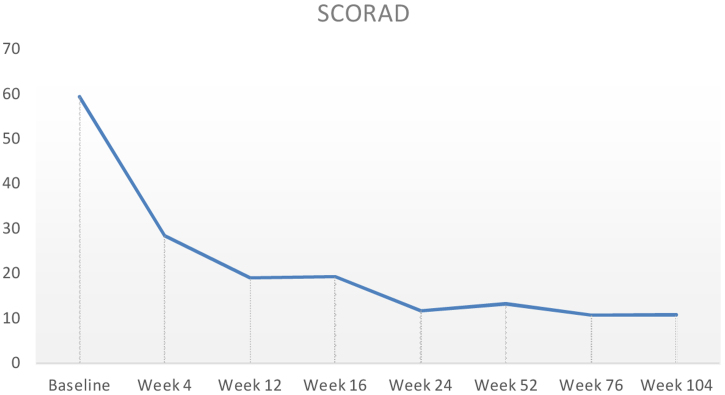
Mean percentage change in Scoring Atopic Dermatitis (SCORAD) from baseline through week 104 in patients treated with dupilumab.

**Figure 2:**
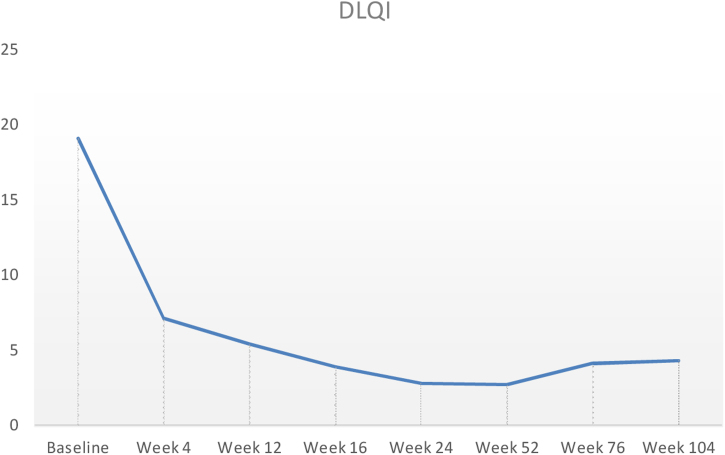
DLQI from baseline through week 104. DLQI, dermatology life quality index.

**Figure 3:**
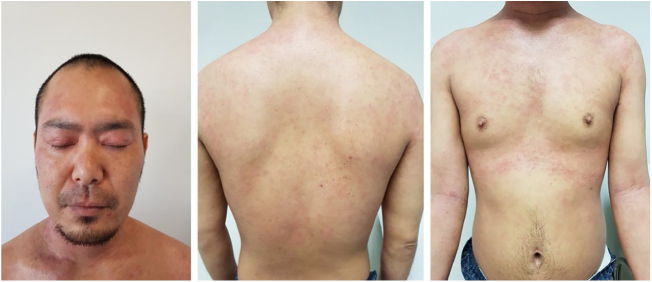
Baseline state on one patient in our series (SCORAD 64).

**Figure 4:**
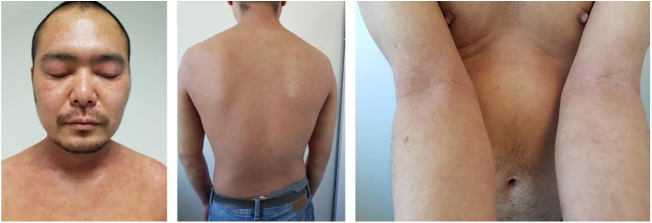
16w after Dupilumab treatment (SCORAD 16).

This retrospective multicentric study on a cohort of 30 patients with severe AD implies the longer follow-up published to date on real-life experience with dupilumab and confirms the efficacy of this agent for refractory cases. A statistically significant reduction in SCORAD was achieved at week 4 and maintained through week 104. The clinical improvement was accompanied by a considerable amelioration of quality of life, as measured by DLQI, and the most bothersome symptom, itch, assessed with pruritus VAS. Dupilumab was well tolerated in most patients, with only 10% of patients suffering from conjunctivitis. These results are aligned with what was recorded in Clinical Trials but contrast with other real-world studies reporting eye symptoms in up to 62% of cases.^5^ From our experience, the use of lipid emulsion eye drops combined with hyaluronic acid eye drops as a prophylactic measure may have a protective effect against dry eyes and eye pruritus, which may have a key role in the development of conjunctivitis Injection-site reactions were not reported by our patients. Limitations of the study are its retrospective nature and the small sample size.

To sum up, our real-life study confirmed that dupilumab is an effective treatment in patients with severe AD and provides the longest follow-up published to date.

## Financial support

None declared.

## Author’s contributions

Jose Juan Pereyra-Rodriguez: Acquisition of data; editing; study concept and design; writing; critical review.

Javier Dominguez-Cruz: Acquisition of data; editing; design; critical review.

Jose Carlos Armario-Hita: Acquisition of data; editing; design; critical review.

Ricardo Ruiz Villaverde: Acquisition of data; editing; study concept and design; writing; critical review.

## Conflicts of interest

None declared.

## References

[1] Silvestre Salvador J.F., Romero-Pérez D., Encabo-Durán B. (2017). Atopic dermatitis in adults: a diagnostic challenge. J Investig Allergol Clin Immunol.

[2] Serra-Baldrich E., de Frutos J.O., Jáuregui I., Armario-Hita J.C., Silvestre J.F., Herraez L. (2018). Changing perspectives in atopic dermatitis. Allergol Immunopathol.

[3] Ruiz-Villaverde R., Dominguez-Cruz J., Armario-Hita J.C., Martinez-Pilar L., Alcantara-Luna S., Pereyra-Rodriguez J.J. (2019). Dupilumab: short-term effectiveness and security in real clinical practice – A retrospective multicentric study. J Eur Acad Dermatol Venereol.

[4] Armario-Hita J.C., Pereyra-Rodriguez J., Silvestre J.F., Ruiz-Villaverde R., Valero A., Izu-Belloso R. (2019). Treatment of moderate-to-severe atopic dermatitis with dupilumab in real clinical practice: a multicentre, retrospective case series. Br J Dermatol.

[5] Ferrucci S., Casazza G., Angileri L., Tavecchio S., Germiniasi F., Berti E. (2020). Clinical Response and Quality of Life in Patients with Severe Atopic Dermatitis Treated with Dupilumab: A Single-Center Real-Life Experience. J Clin Med.

